# Pattern and variation in simple sequence repeat (SSR) at different genomic regions and its implications to maize evolution and breeding

**DOI:** 10.1186/s12864-023-09156-0

**Published:** 2023-03-21

**Authors:** Meiqi Zhao, Guoping Shu, Yanhong Hu, Gangqiang Cao, Yibo Wang

**Affiliations:** 1 Zhengzhou University Graduate Student Training Base at Beijing Lantron Seed, Zhengzhou, 450001 China; 2 School of Agricultural Sciences, Zhengzhou University, Zhengzhou, 450001 China; 3 Center of Biotechnology, Beijing Lantron Seed, Zhengzhou, 450001 China; 4 Henan LongPing-Lantron AgriScience & Technology Co., LTD, Zhengzhou, 450001 China

**Keywords:** Simple sequence repeat (SSR), Long terminal repeat-retrotransposons (LTR-RTs), Maize genome, Evolution, Molecular marker, Breeding

## Abstract

**Background:**

Repetitive DNA sequences accounts for over 80% of maize genome. Although simple sequence repeats (SSRs) account for only 0.03% of the genome, they have been widely used in maize genetic research and breeding as highly informative codominant DNA markers. The genome-wide distribution and polymorphism of SSRs are not well studied due to the lack of high-quality genome DNA sequence data.

**Results:**

In this study, using data from high-quality de novo-sequenced maize genomes of five representative maize inbred lines, we revealed that SSRs were more densely present in telomeric region than centromeric region, and were more abundant in genic sequences than intergenic sequences. On genic sequences, tri- and hexanucleotide motifs were more abundant in CDS sequence and some mono- and dinucleotide motifs were more abundant in UTR sequences. Median length and chromosomal density of SSRs were both narrowly range-bound, with median length of 14-18 bp and genome-wide average density of 3355.77 bp/Mbp. LTR-RTs of < 0.4 Mya had higher SSR density (4498-4992 bp/Mbp). The genome-specific and motif-specific SSR polymorphism were studied. Their potential breeding applications were discussed.

**Conclusions:**

We found that the median length of SSR sequences of different SSR motifs was nearly constant. SSR density in genic regions was much higher than intergenic regions. In addition, SSR density at LTR-RTs of different evolutionary ages varied in a narrow range. The SSRs and their LTR-RT carriers evolved at an equal rate. All these observations indicated that SSR length and density were under control of yet unknown evolutionary forces. The chromosome region-specific and motif-specific SSR polymorphisms we observed supported the notion that SSR polymorphism was invaluable genome resource for developing highly informative genome and gene markers in maize genetic research and molecular breeding.

**Supplementary Information:**

The online version contains supplementary material available at 10.1186/s12864-023-09156-0.

## Introduction

Maize is one of the most important crops. Breeding new varieties more effectively for better commercial values to end users have always been the goal and primary task of maize breeders. Great effort has been made in genetic and genome research to provide molecular markers for breeding and gene discovery for trait development [1–3]. More than 80% of maize genome is composed of repeats of two major classes: the tandem repeat and interspersed repeat; simple sequence repeats (SSRs), as a tandem repeat and accounts for less than 1% of maize genomes whereas LTR (Long Terminal Repeat), as an interspersed repeat, accounts for more than 70% of maize genome.

Simple sequence repeats (SSRs) or microsatellites, are DNA segments with a tandem repeat motif of 1–6 nucleotides [4]. SSRs are divided into mononucleotide, dinucleotide, trinucleotide, tetranucleotide, pentanucleotide and hexanucleotide according to the number of nucleotides in the repeating unit [5]. Although SSRs are usually defined as repeats of 1- to 6-bp motifs [6], heptanucleotide and octanucleotide SSRs have also been involved in some plant researches [7, 8]. Compared with other molecular markers, SSRs are highly polymorphic, codominant and highly informative. SSRs are important tools in maize genetics and breeding [9–11].

In the past, SSRs were considered to be non-functional and therefore subject to little selection pressure, and are evolutionarily insignificant repetitive elements [12]. However, more and more studies have shown that SSR is not only functional but also under various forces of selection. SSR is mainly formed by polymerase slippage during DNA replication resulting in strand mismatches [13]. In addition, the mechanism of the high mutation rate of SSR also includes errors in recombination, unequal exchange, and impaired DNA mismatch repair mechanisms [14]. This polymorphism or variation of SSR may affect genome evolution. In addition to the above mechanisms, there may be other mechanisms that make SSR expand in the genome, which deserves further study.

Previous studies have shown that transposons serve as carriers for SSR expansion, and this type of expansion is still going on in the shrimp genome [15]. Studies also have shown that distribution of SSR on chromosomes is not random, and is closely associated with gene-rich regions in plant genomes [16]. In the soybean genome, SSR markers and genes are more densely distributed near telomeres than near centromeres [17]. Sequence variations and physical location of SSRs can affect gene expression and protein function, for example, SSRs located in UTR and intronic regions may affect mRNA splicing or translation [18]; SSRs can affect transcriptional and post-transcriptional gene regulation. Changes of SSRs in exon increase the incidence of frameshift mutations [19]. SSR can serve as an evolutionary “tuning knob” to quickly adapt to new environments [20]. Studies have shown that tandem repeats are common in many protein sequences, and this mechanism may contribute to the rapid evolution of proteins [21].

Since different species may have evolved different SSR patterns (motif, ratio, and age), studying the SSR patterns at different species might bring different insight to genome evolution of different species [22, 23].

In addition, SSR is widely used as a molecular marker, and SSR is highly polymorphic [3]. Unlike SNP markers, SSR marker polymorphism is variation in numbers of repeats and is expressed as length polymorphism and is easily detected by PCR and gel electrophoresis [24]. SSR markers have been widely used in plant genetic map construction, genetic diversity and variety identification, and molecular marker-assisted breeding [24].

Due to the lack of high-quality genomic sequence data, the distribution of SSR in the genic and intergenic region have not been systematically studied. Eighty percent of maize genome is repetitive sequence, which is a major challenge to DNA sequencing, the NGS sequencing technology is not capable to read the repetitive sequence to build complete reference genome [25]. In recent years, the availability of the de novo third-generation sequencing technologies, that integrates PacBio single molecule real-time sequencing, BioNano optical genome mapping and Hi-C (High-throughput chromosome conformation capture) technologies, have made the high-quality reading and assembly of repetitive sequence to construct a complete maize genome a reality.

Using high-quality de novo-sequenced maize genome data we have examined the pattern and variation of SSRs in different genome regions in five genomes of modern commercial maize inbred lines. We hope our findings would shed light on maize genome evolution, genetic research and molecular breeding.

## Materials and methods

### Genome sequences source

Five maize genomes were selected in this study because of their high de novo sequencing quality and their representativeness in maize heterotic group pattern and germplasm diversity (Table S1). The five inbred lines used in this study represent five major distinct types of the world maize germplasm: B73 and W22 are maize backbone inbred lines from the United States, which belong to the SS (Stiff Stalk) and NSS (Non-Stiff Stalk) heterotic groups, respectively. SK is a Peruvian landrace and is a tropical small-grain maize variety. PE0075 is the representative of European flint maize. LT2357 is a parental inbred line of several big commercial hybrids in China Summer corn belt [26] a major corn commercial production region in China with climate unique from other regions of the world.

Among these genomes, the SK genome was downloaded from National Genomics Data Center (https://ngdc.cncb.ac.cn/), LT2357 was from Beijing Lantron Seed, and other genomes were downloaded from Maize GDB (https://www.maizegdb.org/).

### Genome-wide identification of SSRs

Microsatellite search module (MISA), a SSRs motif scanning tool written in Perl (http://pgrc.ipk-gatersleben.de/misa/), was used for the identification and localization of perfect microsatellites, compound microsatellites and imperfect microsatellites which were interrupted by a certain number of bases [27]. According to MISA, the identified motifs were one to six nucleotides in size, and the minimum repeat unit was defined as 10 for mononucleotides (MNRs), 7 for dinucleotides (DNRs), 6 for trinucleotides (TNRs), 5 for tetranucleotides (TTRs) and 4 for pentanucleotides (PNRs) and hexanucleotides (HNRs). After modifying the corresponding SSR identification related parameters in the misa.ini file, run the command: perl misa.pl genome.fasta. It is important to note that misa.ini and misa.pl need to be placed in one folder.

### Characteristics of SSRs in maize genomes

The SSR sequences detected from genome were divided into 10 groups by length, 10 bp apart, namely 10-20, 21-30, 31-40, 41-50, 51-60, 61-70, 71-80, 81-90, 91-100 and 101+ bp [28]. In the quantity of each length group, and the relationship between the length of SSRs and their quantity were shown in Fig. S1. Python scripts were developed to count the GC-content of all types of SSR.

### SSR distribution in different genomic regions

The protein coding sequence (CDS), untranslated regions (UTR), genic regions (Intron, CDS, UTR) and intergenic regions were extracted from GFF or GTF files of each maize genome. Python scripts were developed to identify SSRs and their quantity and length in the different genomic regions.

### LTR retrotransposons identification and analysis

Since most retroviral genomes, or retrotransposons are flanked by LTR (long terminal repeat), in this study, we focus on retrotransposons with LTR. Full-length LTR-RTs (Long terminal repeat-retrotransposons) were identified in maize genome using software tool: LTR_Finder with the following parameters: ‘-D 20000 -d 1000 -L 7000 -l 100 -p 20 -C -M 0.9’, LTRharvest, and LTR_retriever [29] with default parameters. To calculate the date of insertion of each LTR retrotransposons, the 5′ and 3′ region of the same LTR were aligned using MUSCLE [30]. The “Distmat” utility in the MEGA [31] software package was used to calculate the accumulated difference, or divergence ‘D’ between 5′ and 3′ LTR end. Insertion time (T) of the LTR a retrotransposon was calculated using the formula T = D/2 × r, where ‘r’ is the TE-specific mutation rate of 1.3 × 10^− 8^ per site per year [32]. To study the relationship between LTR-RT and SSR expansion, LTR-RTs were divided into different age groups, using the Python script we developed.

## Results

### Features of six types of SSR motifs in maize genomes

**Fig. 1:**
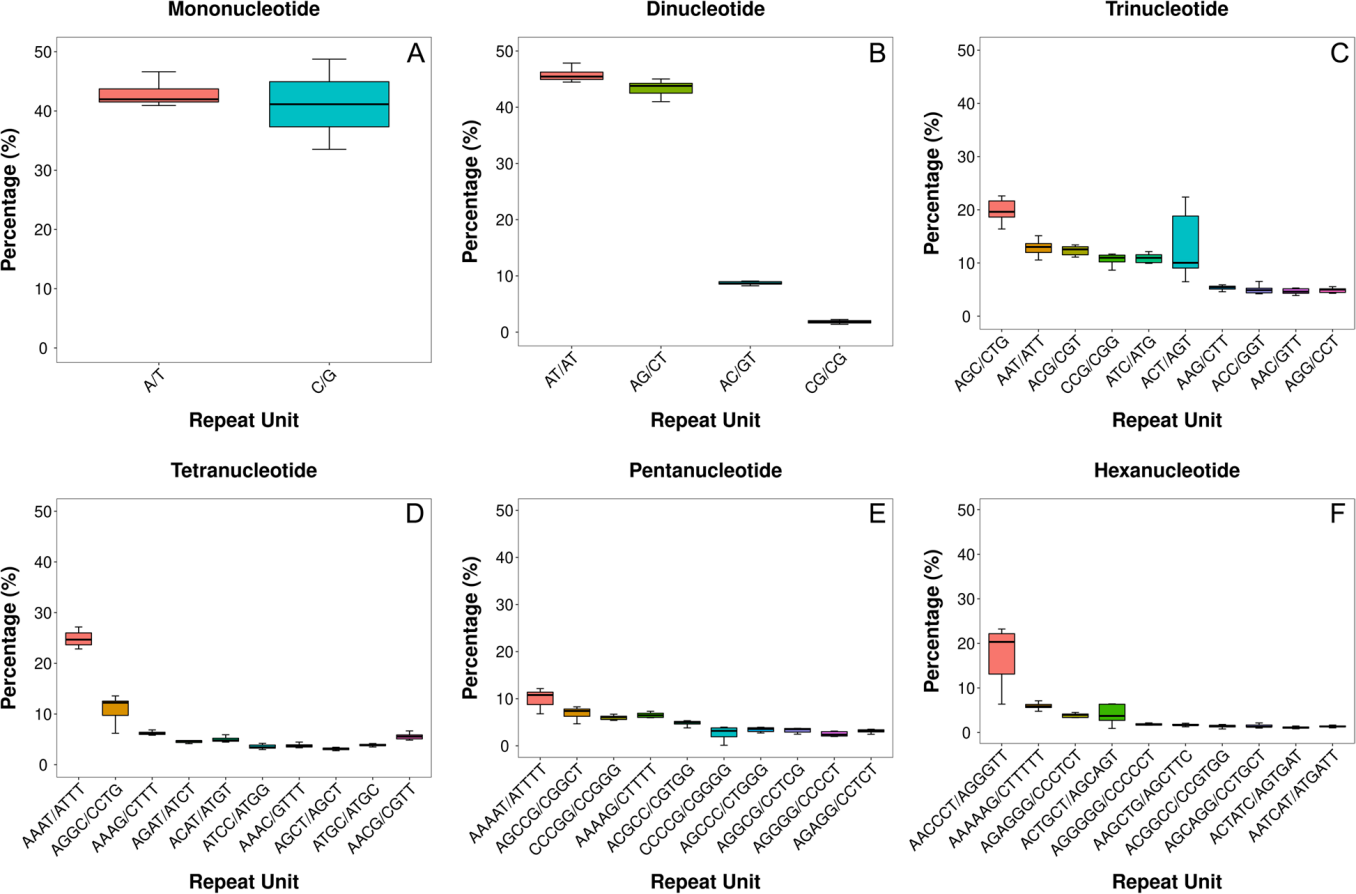
Frequency of different SSR repeat units from Six SSR Motifs. (Percentage refers to the proportion of the number of specific SSRs to the total number of SSRs of six types of nucleotides)

All repeat units of mono-, di-and trinucleotide SSR motifs and the top 10 repeat units of tetra-, penta- and hexanucleotide SSR motifs were collected. Their relative proportion and variation among five maize genomes, referred as genome-specific variation, were plotted using Tukey’s Box-plot (Fig. 1). As shown in Fig. 1A, different repeat units from the same SSR motif could have very large difference in genome abundance and proportion. Different repeat units could have very different genome-specific variation. Four repeat units from four SSR motifs: C/G, ACT/AGT, AACCTT/AGGGTT, and ACTGCT/AGCAGT showed largest genome-specific variation (Fig. 1A, C, F).

**Fig. 2:**
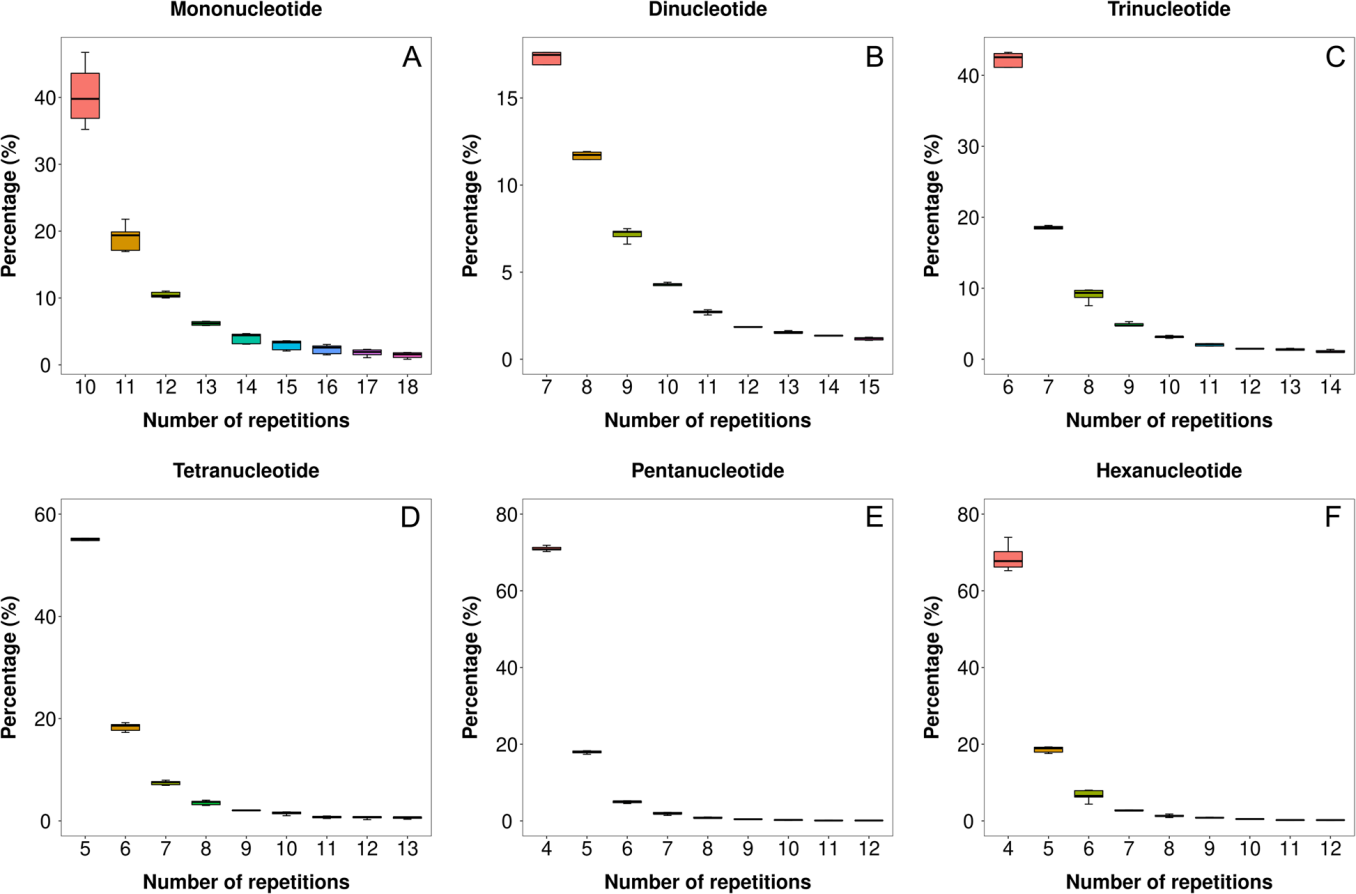
Length Polymorphism of Six nucleotide motif: **A** Mononucleotide, **B** Dinucleotide, **C** Trinucleotide, **D** Tetranucleotide, **E** Pentanucleotide, **F** Hexanucleotide

The length polymorphism of six SSR motifs at Fig. 2 showed that for any SSR motif, the frequency of each repeat unit (which can be viewed as allele in a SSR motif locus) decreased as the number of repeat units increased. It also showed that different motifs had different allele structure, Tetra-, Penta- and Hexanucleotide motifs had the most skew allelic structure and the top two most abundant alleles, which were also the top two shortest in length, accounted for near 80% of total length polymorphism (Fig. 2D, E, F). Whereas the dinucleotide motif had the flattest allele structure and the top two alleles only accounted for near 30% of total polymorphism (Fig. 2B), the mono- and trinucleotide motifs were in between, accounted for near 60% of total polymorphism (Fig. 2A, C).

Table 1 showed that there were 144,756 SSR segments in the maize genome of 2.3 Gb and the mono-, di-, and trinucleotide motif were at least three times as abundant as other three motifs; there was only 5.88% quantity variation among maize genomes on average but there was larger genome variation for mono- (9.47%), trinucleotide (8.0%) and hexanucleotide (13.77%).

**Table 1:** The quantity of six SSR Motifs in five maize genomes

	MNR	DNR	TNR	TTR	PNR	HNR	Total
B73	74,612	41,595	16,223	3484	5259	3222	144,395
W22	63,503	43,239	15,279	3389	4861	2602	132,873
SK	78,951	41,952	16,330	3464	5334	3835	149,866
PE0075	71,105	41,531	16,874	3404	5286	3168	141,368
LT2357	81,236	43,039	18,879	3385	5311	3427	155,277
AVG	73,881	42,271	16,717	3425	5210	3251	144,756
CV (%)	9.47	1.92	8.00	1.33	3.78	13.77	5.88

MNR, DNR, TNR, TTR, PNR, HNR indicate mono-, di-, tri-, tetra, penta- and hexa- nucleotide SSRs

*AVG* Average, *CV* coefficient of variation

Table 2 showed that mononucleotide SSR motif had the highest GC content (61.65%), whereas the dinucleotide and tetranucleotide SSR motifs had the lowest GC content (18.9, 37.16%). The table also showed that genome-specific GC content variation measured by CV of five maize genomes decreased along the increase of nucleotide number of a SSR motif, from 5.98% for mononucleotide to 1.1% for hexanucleotide.

**Table 2:** GC-content of six SSR Motifs in five maize genomes

Genome	GC content (%)					
	Mononucleotide	Dinucleotide	Trinucleotide	Tetranucleotide	Pentanucleotide	Hexanucleotide
B73	64.10	19.36	46.48	36.53	53.50	55.68
W22	55.87	17.18	46.27	37.33	54.98	56.95
SK	65.15	19.46	46.57	38.07	54.26	55.53
PE0075	60.42	18.92	46.17	37.66	53.17	56.04
LT2357	62.72	19.59	42.09	36.23	53.70	55.43
AVG	61.65	18.90	45.52	37.16	53.92	55.92
CV (%)	5.98	5.25	4.23	2.07	1.32	1.10

*AVG* Average, *CV* Coefficient of variation

**Fig. 3:**
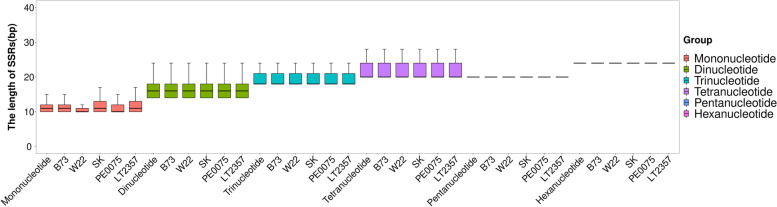
SSR Length polymorphism in six SSR motifs and five maize genomes

Figure 3 showed both the length polymorphism and genome-specific variation for each of the five SSR motifs. Di-, Tri-, and Tetra-nucleotide motif showed large genome-specific variation (wide box and long whisker) whereas Penta-and Hexa-nucleotides showed very little genome-specific variation, all the five motifs showed very little among-genome variation in SSR median length; Mononucleotide motif was unique in that both the median length and the length variation differ among five inbred genomes; inbred genome SK showed long median length and large length variation whereas W22 showed short median length and small length variation. As Table S2 showed the median length of SSRs increased from 11 bp for mononucleotide motif to 24 bp for hexanucleotide motif, and the 1st Qu.-3rd Qu. percentile increased from (10 bp-12 bp) for mononucleotide motif to (24 bp-24 bp) for hexanucleotide motif, however the Tetra and Penta nucleotide motif showed almost the same median length, indicating that the increase in median length is not proportional to the length of repeat unit.

### SSR quantity and density in different genomic regions

**Fig. 4:**
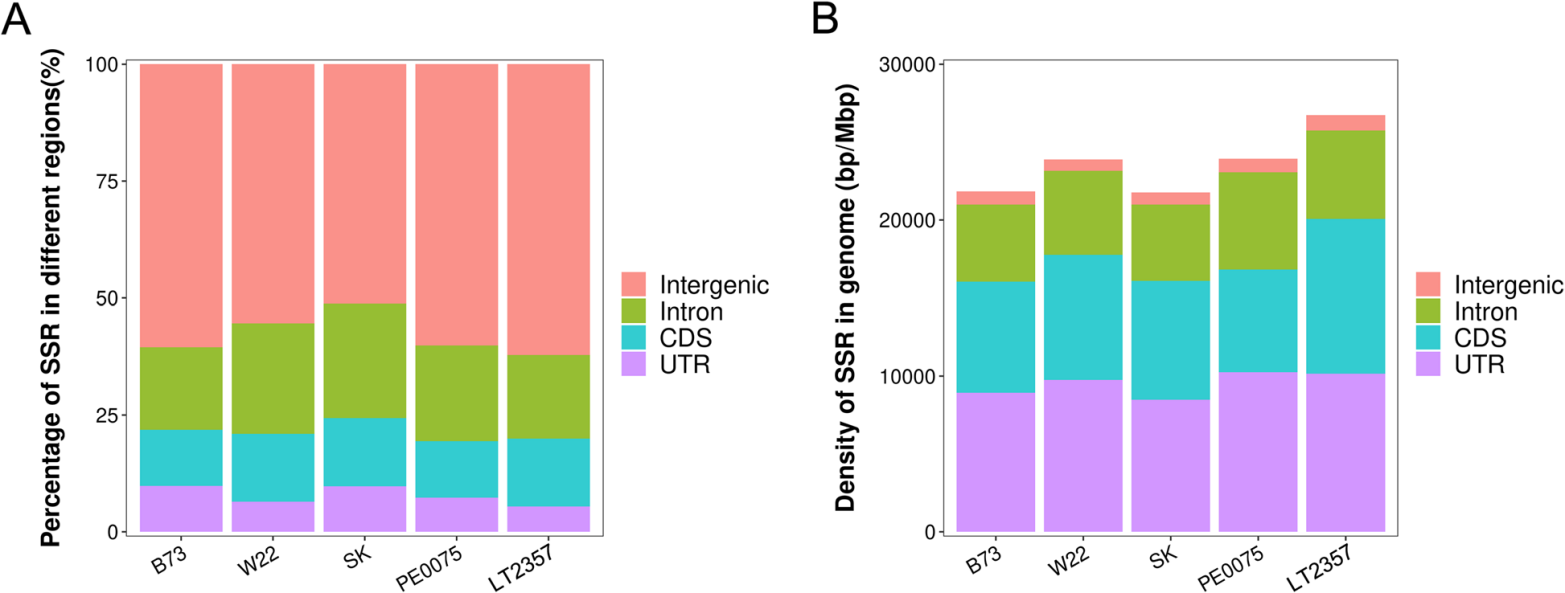
SSR Quantity (**A**) and Density (**B**) in different genomic regions

As Fig. 4A showed, by absolute quantity or count of SSR fragment, intergenic region had more SSR than the genic region (subdivided into Intron, CDS and UTR). In the genic region, Intron has more SSRs than CDS and UTR; However, as Fig. 4B showed, by density calculated as ratio of total length of SSR / length of maize genome, intergenic region had much lower SSR density than genic region. In genic region, the UTR region had higher SSR density than CDS and Intron. Overall, we see one ascending order for SSR quantity: Intergenic region > Intron > CDS > UTR and one descending order for SSR density: UTR > CDS > Intron > Intergenic region. As Table S3 showed, five inbred line genomes had almost the same level of low SSR density in intergenic region (731.96 bp/Mb - 989.29 bp/Mb), but had 6 to 9 folds higher SSR density in Intron, CDS and UTR regions (4964.29 bp/Mb - 10,256.20 bp/Mb).

**Fig. 5:**
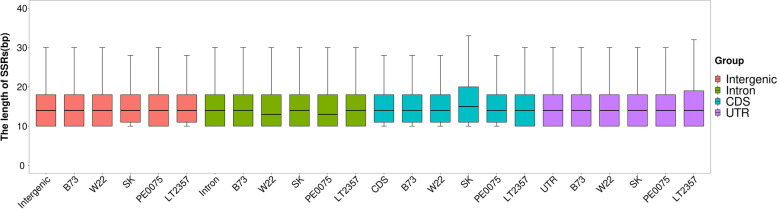
The among-genome SSR length polymorphism in different genomic regions

Figure 5 showed SSR length polymorphism among five inbred line genomes using box plot; the SK had smaller among-genome polymorphism in intergenic region but larger among-genome polymorphism in CDS region, LT2357 had smaller among-genome polymorphism in intergenic region and larger among-genome polymorphism in UTR region, but B73 showed no big difference in among-genome polymorphism in all four different genomic regions. Table S4 showed that the length of SSR segments was narrowly range bounded, with median of 14 bp and 1st Qu. - 3rd Qu. percentile 10-20 bp for Intergenic, Intronic, UTR region and median of 14-16 bp and 1st Qu. - 3rd Qu. percentile of 11-20 bp for Intergenic, Intronic, UTR region.

**Fig. 6:**
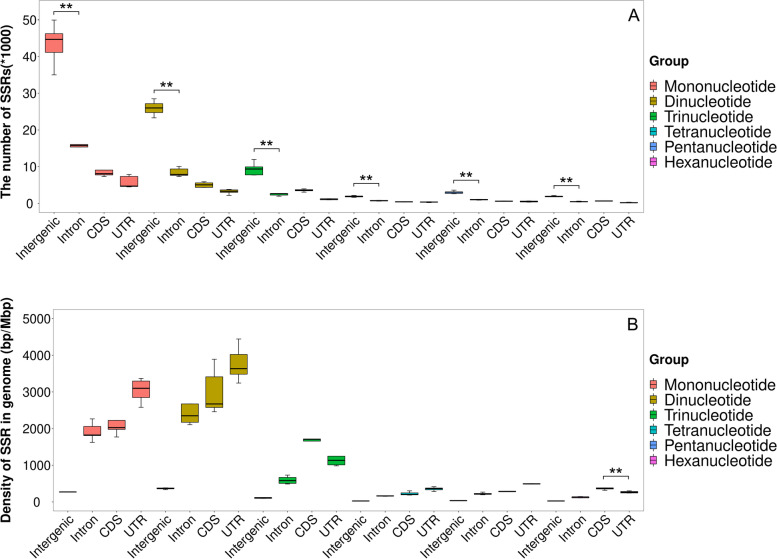
The Quantity (**A**) and Density (**B**) of five SSR motifs in four genome regions (**P < 0.01)

For all six SSR motifs, Fig. 6 showed that the intergenic region had the largest quantity (Fig. 6A) but had the lowest density measured using length ratio (bp/Mbp) (Fig. 6B). Among the three genic regions, UTR region had the smallest SSR quantity (Fig. 6A) but the highest SSR density for all SSR motifs except for trinucleotide and hexanucleotide motif; the CDS region had the highest SSR density for trinucleotide and hexanucleotide (Fig. 6B). Figure 6B also showed that the dinucleotide motif had large genome-specific variation in CDS and Intron. Both mononucleotide and dinucleotide motifs had large genome-specific variation in UTR region.

To further examine the genome region-specific difference in SSR distribution, from Fig. 1, eight SSR repeat units were selected from different SSR motifs that showed either large genome-specific variation or large allele frequency: (A/T)n, (C/G)n, (AT/AT)n，(AG/CT)n, (ACT/AGT)n, (AAAAT/TTTTA)n, (AACCCT/AGGGTT)n and (ACTGCT/AGCAGT)n. Their partition patterns are shown at Fig. S2, both trinucleotide and hexanucleotide had the highest density at CDS region than at all other genome regions whereas the mononucleotide (A/T)n and (C/G)n and dinucleotide (AG/CT)n showed the highest density at UTR region.

### The chromosome pattern of genes, SSRs, and LTR-RTs

**Fig. 7:**
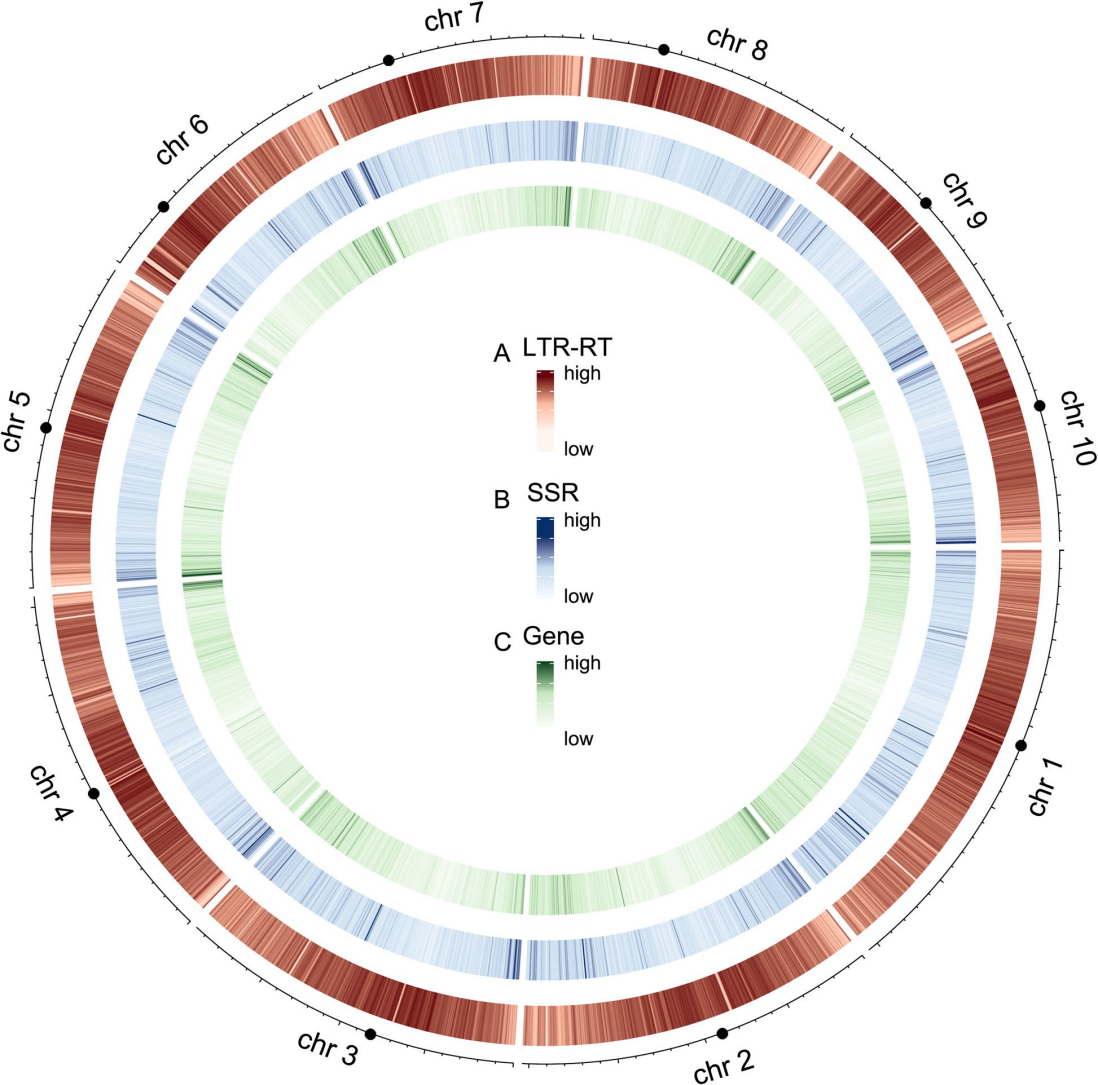
The chromosomal distribution of LTR-RT (**A**), SSR (**B**), and gene (**C**) in the B73 genome. (The black dot in the outermost circle represents the centromere)

As the Circos plot at Fig. 7 showed, in B73 genome, LTR-RT sequences were more abundant in the centromeric region. SSR sequences and genic sequences were more abundant at both ends of chromosomes near telomeres. On almost any region at both ends of chromosomes, we observed the presence of SSRs and genes and the absence of LTR-RT, which took place together or the reverse was also true, indicating that the density of SSRs was higher at gene-rich region than at LTR-RT-rich region. It was worth mentioning that the chromosome distribution of repetitive sequences and genes in W22, SK, PE0075 and LT2357 genomes (Fig. S4) were also similar to those in B73.

### Dynamic changes of SSR carried by LTR-RT of different evolution ages

**Fig. 8:**
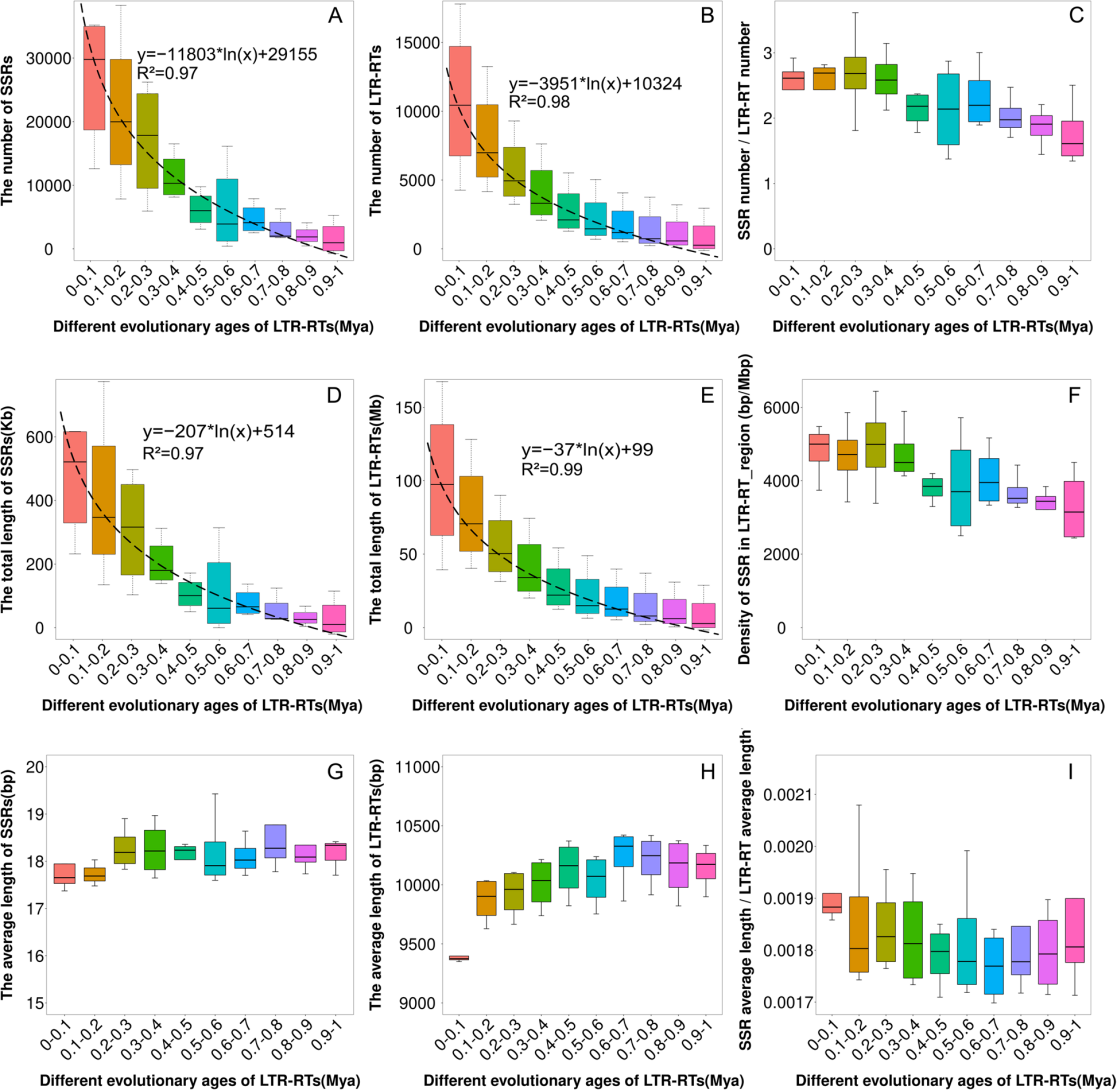
Change in length and density for LTR-RT and SSR in 0.0-1.0 Mya. **A** Change in quantity (the number of SSRs) carried by LTR-RTs of different evolution ages (1 Mya = 1 million years ago). **B** Change in quantity (the number) of LTR-RTs at different evolution ages. **C** Change in SSR density (as the number ratio of SSRs / LTR-RTs). **D** Change in the total length of SSRs carried by LTR-RTs. **E** Change in the total length of LTR-RTs. **F** Change in SSR density in LTR-RT region. **G** Change in the average length of SSRs. (H) Change in the average length of LTR-RTs. **I** Change in the average length of SSRs / LTR-RTs. The dotted line in the figures represent the ln trend line

We firstly divided the LTR-RTs into different evolution age groups and then counted the SSR quantity and calculated the SSR density for each LTR-RT age group. We saw a negative association between SSR quantity and the age of LTR-RT where the SSRs reside (Fig. 8A and B), that is, the younger the age group, the larger the SSR quantity, the negative association between the quantity and age can be fitted by a log regression curve: y = −b*ln(x) + a. A similar negative association was also observed between total length and age (Fig. 8D and E). The negative association between LTR-RT age and SSR quantity was particularly strong among the four age groups younger than 0.4 Mya. Compared the regression curves of Fig. 8A and B, it was clear that SSR quantity and the LTR-RT quantity change at similar rate, Fig. 8C showed that the SSR density did not change much among the four LTR-RT age groups of less than 0.4 Mya, either measured by number ratio (2.6-2.7 SSR segments per LTR segment, Fig. 8C) or by length ratio (4498-4992 bp/Mbp, Fig. 8F); A slow descending of SSR density was observed in the LTR-RT groups of older than 0.4 Mya (Fig. 8C, F). Figure 8G, H showed that the average length of both SSR and LTR-RT were narrowly range bound across all LTR-RT age groups, ranging from 17.65-18.37 bp per segment for SSR and 9.37 kb-10.33 kb per segment for LTR-RT respectively, and no trend was observed (Fig. 8G, H); however, the SSR density as the length ratio of SSR/LTR-RT was about 0.001769 - 0.001883, nearly constant across all age groups (Fig. 8I).

## Discussion

### SSR quantity and density in genic and intergenic region

Although overall we saw more SSRs in intergenic region than genic region, we have found that the genic regions had much higher SSR density, at least 11, 6, and 9 times higher in UTR, Intron, and CDS region than intergenic region respectively. Studies in other organisms have also shown that the distribution of SSR markers on chromosomes are closely related to gene-rich regions in the plant genomes [16]; in soybean genome, there are more SSR markers and genes near the telomere region than the centromere region [17].

Among different genic regions, our data have shown that the CDS region had higher quantity and density of trinucleotide SSR motif and its duplicated version, hexanucleotide SSR motif, than other SSR motifs. It has known for long that protein coding sequence or CDS are under strong selection pressure and any insertion/deletion of nucleotide segment not equal to the multiple of 3 at CDS region would more likely cause a frame-shift are thus deleterious, and the insertion of the trinucleotide and hexanucleotide at CDS region are more likely lead to one or two amino acid replacement and thus to be more tolerable in genome evolution. The preferred presence of trinucleotides in CDS region have been shown as an evolutionary mechanism of protecting genes from being affected by frameshift mutations [33]. Studies have shown that tandem repeats are common in many protein sequences, SSR may be used as an evolutionary tuning knob to enable an organism quickly adapts to the new environment [20] and may provide a source of mutation for the rapid evolution of protein structure and function [21]. Our data also have shown that mononucleotide A/T was most abundant at UTR region. Previous researches have suggested that the poly(A) tails of densely scattered retroposed sequences and processed pseudogenes are responsible for this higher proportion of A/T-rich repeat sequences observed [13]. Traditionally, SSRs are considered to be non-functional and hence neutrally evolving because of low selection pressure [12]. However, more and more data from recent studies have shown that SSRs not only affect the protein coding, but also can affect the expression of genes in transcription and post-transcriptional regulatory networks [18, 19].

### The length and density conservation in maize SSRs

We have found that the median length of 14-18 bp held well for SSRs at both genic and intergenic regions and at all age groups of LRT-RTs. We also found that the chromosomal density of SSR was narrowly range-bound, with genome-wide average density of 3355.77 bp/Mbp. LTR-RTs of < 0.4 Mya were found to have higher but nearly constant SSR density of 4498-4992 bp/Mbp. Our data supported the notion that both the length and density of SSR were under control of biological and evolutionary forces. DNA polymerase slippage model and unequal chromosome cross-over model and other mechanisms are widely recognized [13, 14, 26] as major biological forces of producing SSR length polymorphism. Similar observations have also reported in other researches [28, 34, 35]. However, maintaining SSR as 14-18 bp segments required constant trimming or removal of segments much longer than 18 bp, the underlying mechanism is unknown but intriguing. It has been widely speculated that SSRs with longer sequence are subjected to greater purifying selection pressure and are more likely to be removed. The SSRs with shorter lengths are retained, the length extension and contraction take place constantly until a stable length threshold is reached. In most eukaryotes a threshold of up to 20 bp are reported [36, 37]. We are currently studying the evolutionary forces controlling SSR density in maize genomes.

### Evolution of SSR and LTR-RT at equal rates

LTR-RTs are abundantly present in maize genome, and as autonomous transposons, they can self-amplify and move (duplicated by copy and paste). We showed that LTR-RTs carried a large number of SSRs and the SSR density differs in LTR-RT of different age. In the LTR-RT groups younger than 0.4 Mya (Fig. 8), we saw a rapid increase (LTR-RT expansion) in quantity of LTR-RT and the increase of SSR quantity at a similar rate and nearly constant SSR density. We call this phenomenon as “evolution at equal rates” of SSR and its LTR-RT carrier. The underlined mechanism that leads to evolution at equal rates and nearly constant SSR density we observed remains unclear. One possible mechanism is genetic hitchhiking-like effect, genetic hitchhiking was first proposed by Smith and Heigh in 1974 [38] to explain the selection sweep observed in chromosome region near a mutation site under strong positive selection, and have been widely observed in various species [39, 40]. In our case, a SSR sequence residing on the LTR-RT region might behave like a hitchhiker and the LTR-RT serves as a carrier; thus, the rapid increase or expansion of LTR-RTs in the age groups of less than 0.4 Mya naturally leaded to the increase or expansion of SSRs (as riders) at the same rate, which explained why the density of SSR on LTR-RT (Fig. 8C and F) (obtained by divided or normalized by LTR-RT) was nearly constant (or narrowly range-bound) at four age groups less than 0.4 Mya. It is known that SSR is propagated by chromosome replication errors caused by DNA polymerase slippage and the DNA mismatch repair (MMR) during chromosomal duplication [41–44], our observation suggested that “the hitchhiker-like effect” might be a second mechanism of SSR expansion. The expansion of both SSR and LTR-RT is slow in age groups older than 0.4 Mya, SSR density in these groups are also low and descend with age. Further researches are needed to verify the existence of this hitchhiking-like mechanism of SSR expansion. Many researches have shown that transposons or TEs have played very important roles in maize genome evolution [45]. The involvement of TEs in SSR origin and expansion have been reported in rice [46], lepidopteran insects [47], penaeid [4], and snakes [2]. It is also known that *Alu* and *L1* transposable elements in the human genome play a direct role in the creation and propagation of SSRs [22]. Our results were also consistent with the study of penaeid shrimp genome, which showed that transposons are the carrier of SSR expansion, and this expansion was still carried out in the penaeid shrimp genome [15].

### Length polymorphism and molecular breeding

SSR is a very important DNA molecular marker in plant breeding and widely used in crop molecular breeding, parentage identification, variety genuineness or authenticity identification and variety intellectual property protection [10, 11, 48–51]. Unlike other types of molecular markers, such as RFLP and SNP, SSR polymorphism is variation in number of repeat units and thus is DNA segment length or DNA molecule size variation. This feature makes its utilization as molecular marker very convenient; the DNA allele polymorphism can be observed by using low-cost laboratory techniques like Agrose gel or PAGE gel electrophoresis, unlike RFLP markers that require restriction enzyme digestion and SNP markers that require florescent labeling. In this study we have identified 144,756 polymorphic SSR segments in the sample of five maize genomes, the finding suggests that SSR polymorphism is sufficient for most genetic research and molecular breeding. Because it is DNA segment length variation, and it is multi-allelic and is much more informative than a bi-allelic SNP marker although the latter is much large by quantity in maize genomes [10, 26]. The genome-region-specific polymorphism we observed, such as, more abundant presence of tri- and hexanucleotide SSR motifs in CDS region, and more abundant presence of mononucleotide SSR motif in UTR region, would provide valuable information for genome region-specific SSR marker design in genetic research and molecular breeding. The finding that SSR density is higher in CDS allows users to design CDS-specific SSR marker to better distinguish closely related species or because the similarity of CDS regions between different varieties of the same species is high. The motif-specific polymorphism we observed, such as flat and skew allele structure of different motif reported in Fig. 2, would help the breeders to select SSR markers with a proper allele structure. The distribution pattern of SSR along different chromosomal regions reported would help researchers tailor a set of SSR markers to represent a particular genome region or an entire genome.

We have found that both mononucleotide and dinucleotide SSR motifs had the largest quantity in maize genome. They also had the largest genome-specific variation in genic region (Fig. 6B). However, we have found that the two motifs had quite different features in other aspects, for instance, the mononucleotide SSR motif had the most skewed allele structure whereas dinucleotide had the flattest allele structure (Fig. 2), and the mononucleotide motif had much higher GC content than the dinucleotide SSR motif (Table 2). Researches have shown that DNA sequences with high GC content will increase the probability of replication slippage, and cause insertion/deletion in DNA amplification [52]. Therefore, our findings and results from literature reports suggest that dinucleotide motif was more suitable than mononucleotide motif to be used to develop molecular markers. In fact, this suggestion has support from maize breeding practice; we have examined the SSR repeats in the 40 standard SSR markers recommended by National Standard of Variety Genuineness and Variety Intellectual Property Protection of China (VGVIP National Standard in short), which were widely used by breeding companies and research institutes in China and found that none of the 40 markers contain mononucleotide repeats, but 28 of them contain dinucleotide repeats. We also found that the 28 dinucleotide markers were mostly TA, GA, CT, AG, TC repeats [48, 49]. Our data has already shown that these five repeats are the most abundant dinucleotide repeats in maize genome (Fig. S3A, B).

In this study, we analyzed the distribution and evolution of SSR in maize genome, and preliminarily discussed the relationship between LTR-RT and SSR. With the progress of sequencing assembly and bioinformatics analysis methods, the reference genomes of many plants have been released. Not only in maize, SSR and other repeats in other species are still worthy of our in-depth study. SSR is no longer the “non-functional sequence” previously thought, nor is it a field difficult to explore by bioinformatics methods. SSR is widely used in genetic analysis of maize and other species. Studying the distribution and evolution of SSR is conducive to the detailed exploration of the potential function of SSR in maize and other species in the future.

## Supplementary Information

**Additional file 1: Table S1.** De novo Sequenced Maize Genomes used in the study.

**Additional file 2: Figure S1.** Length variation of simple sequence repeats (SSRs) in different Maize genomes.

**Additional file 3: Table S2.** SSR Length polymorphism in six SSR motifs and five maize inbred genomes (The same data set for Fig. 2).

**Additional file 4: Table S3.** SSR Quantity and Density in four different genome regions for 5 maize inbred genomes (The same data set for Fig. 3).

**Additional file 5: Table S4.** Length Polymorphism of SSR in different genomic regions among five inbred genomes. (The same data set for Fig. 4).

**Additional file 6: Figure S2.** The density of eight representative SSR repeat units in different genomic regions.

**Additional file 7: Figure S4.** The chromosomal distribution of LTR-RT, SSR, and gene in W22, SK, PE0075, LT2357 genomes. (The black dot in the outermost circle represents the centromere.).

**Additional file 8: Figure S3.** Distribution of the number (A) and length (B) of dinucleotide SSRs with specific repeat units in different maize genomes.

**Additional file 9:** Script for analyzing GC content of SSR.

**Additional file 10:** The SSR identification script in different genomic regions.

## Data Availability

The SK genome can be downloaded in National Genomics Data Center (https://ngdc.cncb.ac.cn/). The B73, W22 and PE0075 genome can be downloaded in Maize GDB (https://www.maizegdb.org/). The LT2357 genome was unpublished data from Beijing Lantron Seed and was uploaded to the NCBI database (PRJNA894302). The datasets generated and/or analyzed during the current study are available from the corresponding author on reasonable request.
