# Supplementary material for: Pattern and variation in simple sequence repeat (SSR) at different genomic regions and its implications to maize evolution and breeding
Source: BMC Genomics. 2023 Mar 21;24:136. doi: 10.1186/s12864-023-09156-0 (PMC10029318; doi:10.1186/s12864-023-09156-0)
Supplement: Supplementary file 9 — Additional file 9: Script for analyzing GC content of SSR. [file 12864_2023_9156_MOESM9_ESM.docx]

### Script 1 Script for analyzing GC content of SSR

with open("B73_SSR_MISA_GC_percent.txt", 'a') as fw:

with open("B73_SSR_MISA_modify", 'r') as f:

for line in f:

line = line.strip("\n")

#get the ssr unit sequence

seq = line.split('\t')[0]

#get the ssr unit length

len_seq = len(seq)

#get the G or C count in the ssr unit

G_count = seq.count("G")

C_count = seq.count("C")

#caculate the GC content

GC_percent = str((G_count+C_count)/len_seq*100)+"%"

rec = str(line) + "\t" + str(len_seq) + "\t" + GC_percent + "\n"

fw.write(rec)

print("done")

f.close()

fw.close()

## B73_SSR_MISA_modify input file format reference:

The first column is the repeating unit, the second column is the number of repetitions, and the third column is the ID of SSRs.

TA 7 ID=1.2

C 25 ID=1.3

G 10 ID=1.4

GA 8 ID=1.5
