# Supplementary material for: Pattern and variation in simple sequence repeat (SSR) at different genomic regions and its implications to maize evolution and breeding
Source: BMC Genomics. 2023 Mar 21;24:136. doi: 10.1186/s12864-023-09156-0 (PMC10029318; doi:10.1186/s12864-023-09156-0)
Supplement: Supplementary file 10 — Additional file 10: The SSR identification script in different genomic regions. [file 12864_2023_9156_MOESM10_ESM.docx]

### Script 2 The SSR identification script in different genomic regions

(Taking SSR identification of genic region and intergenic region in SK genome as an example.)

ssr_bed = 'SK_ssr_misa.gff'

gene_bed = 'SK_gene.gtf'

ssr_in_gene = []

#If the start or end site of a ssr in gene region, this is a 'ssr_in_gene'

with open(ssr_bed, 'r') as f1:

for line in f1:

line = line.strip('\n')

ssr_chr = line.split('\t')[0]

ssr_f = line.split('\t')[3]

ssr_r = line.split('\t')[4]

with open(gene_bed, 'r') as f2:

for line2 in f2:

line2 = line2.strip('\n')

gene_chr = line2.split('\t')[0]

gene_left = line2.split('\t')[3]

gene_right = line2.split('\t')[4]

if gene_chr == ssr_chr:

if gene_left <= ssr_f <= gene_right or gene_left <= ssr_r <= gene_right: #gene_left <= ssr_f <= gene_right or

ssr_in_gene.append(line)

ssr_in_gene = set(ssr_in_gene)

f2.close()

f1.close()

# Write the SSR in gene region to a file

with open('ssr_in_gene.gtf', 'a')as f3:

for i in ssr_in_gene:

i = i + '\n'

f3.write(i)

f3.close()

# Write the SSR in intergenic region to a file

with open('ssr_in_gene.gtf', 'r') as f5:

ssr_in_gene = set(f5.readlines())

print(len(ssr_in_gene))

with open(ssr_bed, 'r') as f4:

all_ssr = f4.readlines()

print(len(all_ssr))

ssr_in_intergenic = [item for item in all_ssr if not item in ssr_in_gene]

print(len(ssr_in_intergenic))

with open('ssr_in_intergenic.gtf', 'a') as f6:

for item in ssr_in_intergenic:

f6.write(item)

f6.close()

# If you need to identify SSRs in other areas, just replace the ' SK_gene.gtf ' file with another bed file.
